# Single-Cell Transcriptomic Analysis Reveals a Tumor-Reactive T Cell Signature Associated With Clinical Outcome and Immunotherapy Response In Melanoma

**DOI:** 10.3389/fimmu.2021.758288

**Published:** 2021-11-05

**Authors:** Min Yan, Jing Hu, Yanyan Ping, Liwen Xu, Gaoming Liao, Zedong Jiang, Bo Pang, Shangqin Sun, Yunpeng Zhang, Yun Xiao, Xia Li

**Affiliations:** ^1^ College of Bioinformatics Science and Technology, Harbin Medical University, Harbin, China; ^2^ Key Laboratory of High Throughput Omics Big Data for Cold Region’s Major Diseases in Heilongjiang Province, Harbin Medical University, Harbin, China

**Keywords:** tumor reactivity, tumor-infiltrating T cells, immunotherapy, exhausted T cells, melanoma

## Abstract

The infiltration of tumor-reactive T cells in the tumor site is associated with better survival and immunotherapy response. However, tumor-reactive T cells were often represented by the infiltration of total CD8+ T cells, which was confounded by the presence of bystander T cells. To identify tumor-reactive T cells at the cancer lesion, we performed integration analyses of three scRNA-seq data sets of T cells in melanoma. Extensive heterogeneous functional states of T cells were revealed in the tumor microenvironment. Among these states, we identified a subset of tumor-reactive T cells which specifically expressed tumor-reactive markers and T cell activation signature, and were strongly enriched for larger T cell receptor (TCR) clones. We further identified and validated a tumor-reactive T cell signature (TRS) to evaluate the tumor reactivity of T cells in tumor patients. Patients with high TRS scores have strong immune activity and high mutation burden in the TCGA-SKCM cohort. We also demonstrated a significant association of the TRS with the clinical outcomes of melanoma patients, with higher TRS scores representing better survival, which was validated in four external independent cohorts. Furthermore, the TRS scores exhibited greater performance on prognosis prediction than infiltration levels of CD8+ T cells and previously published prognosis-related signatures. Finally, we observed the capability of TRS to predict immunotherapy response in melanoma. Together, based on integrated analysis of single-cell RNA-sequencing, we developed and validated a tumor-reactive-related signature that demonstrated significant association with clinical outcomes and response to immunotherapy.

## Introduction

Cancer immunotherapies by immune checkpoint blockade (ICB) aim to reactivate the T cell responses to kill tumor cells. The canonical targets of ICB therapy are cytotoxic T lymphocyte-associated antigen 4 (CTLA4) and programmed cell death protein 1 (PD1). Both treatments eventually depend on the activity of tumor infiltration T cell pool to achieve tumor elimination (1). Compared to conventional cancer therapies, ICB therapies have resulted in durable response in tumor patients. However, the response to ICB treatments vary among patients, only about one-third of patients benefit from immunotherapy in most cancer types (2). It has long been known that multiple factors can affect the effectiveness of ICB, such as tumor mutation burden, expression level of PD-L1, interferon signaling and T cell infiltration (3). Among these, the levels of cytotoxic T cell infiltration of tumor were widely associated with patient prognosis in many cancer types (4), with higher T cell infiltration associated with better clinical outcome. Especially, the presence of active T cells is associated with increased disease-free survival and/or overall survival in human melanoma (5).

Tumor infiltration of CD8+ T cells are primed in lymph nodes and migrated *via* blood to the tumor site, where they exert their effector functions. During the process, T cells received numerous signals and different tumor-specific antigens (TSAs) that influenced their states and functions. Therefore, many single cell researches revealed the high diversity of tumor-infiltrating T cell states in various human cancer types, including melanoma (6), head and neck (7), breast (8), colorectal (9), pancreas and lung cancer (10). Meanwhile, recent literatures have validated that only a proportion of infiltrating T cells that reside in the tumor microenvironment are able to recognize TSAs or tumor associated antigens (TAAs), which we called tumor-reactive T cells (11, 12). Contrarily, there exist another type of infiltrating T cells called bystander T cells, which have been shown to reactive against viruses-related antigens and recognize a range of epitopes unrelated to tumor cells (13). Previous studies have revealed that the efficacy of ICB immunotherapies is dependent on the subset of tumor-reactive T cells with tumor-reactive T cell receptor (TCR) repertoire rather than the bystander T cells (14). However, there is a lack of powerful signatures to efficiently identify tumor-reactive T cells and further to indicate clinical outcomes and response to immunotherapies of tumor patients.

In this study, we utilized scRNA-seq profiles of CD8+ T cells in melanoma to derive a cluster of tumor-reactive T cells, and further developed a tumor-reactive signature (TRS) to indicate the tumor reactivity of tumor samples. We validated the ability of distinguishing tumor-reactive cells or cell groups of the TRS in multiple cohorts. Furthermore, we demonstrated significant correlation of the TRS with clinical outcomes and response to immunotherapy of melanoma patients.

## Materials and Methods

### Data Collection and Processing

Three scRNA-seq datasets of melanoma patients were downloaded from GEO database under accession numbers GSE72056 (6), GSE115978 (15) and GSE120575 (16). For GSE120575, we extracted expression profiles of candidate T cells in the clusters G5-G11. For GSE115978 and GSE120575, candidate T cells were extracted according to the cell labels (“T.CD4”, “T.CD8” and “T.cell”) defined in the original studies. We further investigated the expression distribution of CD8 (average expression of CD8A and CD8) and CD4 in these T cells, and retained only CD8+ T cells for subsequent analysis, which were defined as CD8 > 2 and CD4 < 2 (**Figures S1A-C**). Then we integrated all these CD8+ T cells through the CCA algorithm (17) implemented in Seurat (18). The standard workflow of cell clustering in Seurat was utilized to identify distinct groups of cells based on the integrated data. In brief, PCA was performed on the scaled data, and the top 30 PCs were used for graph-based clustering to identify cell clusters. Cluster-specific genes were identified using the FindAllMarkers function in Seurat based on the “RNA” assay. All datasets used in this study were listed in **Table S1**.

### TCR Analysis

The T cell receptors (TCR) sequences of single cells were kindly provided by the corresponding author of the original study (16). A total of 3078 cells with TCR sequences were used for the TCR analysis. Each unique alpha-beta sequence pair was defined as a clonotype, and the number of cells harboring the same clonotype was calculated as the clonal size. If one clonotype was present in at least three cells, cells harboring this clonotype would be considered as clonal. The within-sample diversity score of TCR repertoire (clonality score) was evaluated by the downsampling-based Shannon’s entropy to correct for differences in repertoire size, through utilizing the iNEXT R package.

### Identifying Tumor-Reactive CD8+ T Cell Signature

In order to characterize tumor-reactive T cells and explore the relationship between tumor reactivity and patient clinical outcome, we developed the tumor reactive signature (TRS). The clusters C1, C2 and C5 all had the characteristics of exhaustion and tumor reactive phenotype. However, C2 and C5 were strongly interfered by cell cycle and IFNG signals, respectively, which were considered as confounding factors of T cell status in the previous studies (19, 20). Therefore, we only used the specifically expressed genes of cluster C1 as the candidate tumor-reactive signature genes through Wilcoxon rank-sum test with false discovery rate adjusted p values (FDR) < 0.05. Then we performed receiver operating characteristics (ROC) analysis for each gene to measure its ability to discriminate cluster C1 from the remaining clusters. Each highly expressed gene was treated as a predictor, and cells inside and outside of cluster C1 was treated as the positive and negative sets, respectively. The tumor-reactive signature genes (TRS) were extracted as the top 20 genes with highest AUC.

### Signature Validation Across Different Cancer Types and Protocols

To validate the tumor-reactive T cell signature, we downloaded 4 scRNA-seq datasets of different cancer types (including hepatocellular carcinoma, non-small cell lung cancer, colorectal cancer and melanoma) from the GEO database under accession numbers GSE98638, GSE99254, GSE108989 and GSE123139, respectively. For the Smart-seq2 datasets (i.e., GSE98638, GSE99254 and GSE108989), we downloaded the TPM-normalized expression profiles. Then we extracted CD8+ T cells in tumor samples, and retained genes with average expression greater than 0.5 and detection rate greater than 0.1. For the MARS-seq dataset GSE123139, we downloaded the raw count profiles. Then we retained CD8+ T cells with number of expressed genes between 500 and 3000, and retained genes with detection rate higher than 0.01. Meanwhile, we obtained 4 bulk datasets containing tumor-reactive T cells or cell groups from the GEO database under accession numbers GSE114944, GSE132810, GSE141878 and GSE145596. For the microarray dataset GSE114944, we downloaded the processed probe expression matrix file and converted it to gene expression profile. For datasets GSE132810 and GSE141878, we downloaded raw count profiles, and for GSE145596 we downloaded the TPM-normalized expression profiles for subsequent analysis. The tumor-reactive T cell signature score for each sample was calculated by using gene set variation analysis (GSVA) (21). We implemented GSVA with default parameters (kcdf=“Gaussian”, min.sz=1) to calculate the TRS scores for all microarray datasets and for RNA-seq datasets with TPM-normalized expression profiles. For RNA-seq datasets with raw count profiles (i.e., GSE132810 and GSE141878), we set the parameter kcdf as “Poisson”. In addition, we also used ssgsea (22), zscore (23) and plage (24) with default parameters to calculate the TRS scores. For single-cell data, we calculated the TRS scores using AUCell (25) and Vision (26) with default parameters. Global significance of differences across multiple groups was evaluated by Kruskal-Wallis test. The Wilcoxon rank-sum test was used to assess the statistical difference between tumor-reactive group and others.

To further validate the correlation between T cell infiltration proportion and the tumor-reactive signature score, we obtained 7 datasets of melanoma, including 4 microarray datasets from the GEO database (GSE22153, GSE65904, GSE19234, and GSE53118), one cohort (Allen2015) kindly provided by the corresponding author (27), one cohort from the ENA database under accession ERP105482 (28), and the TCGA-SKCM cohort from TCGA database. For the four microarray datasets, we downloaded the processed probe expression matrix files and then converted to gene expression profiles. For GSE22153, we also implemented k-nearest-neighbor imputation for missing expression values using the impute R package (29). For the ERP105482 cohort, we used kallisto (30) to quantify gene expression which were then converted to TPM and log2-transformed as described in our previous study (31). The expression profile of the TCGA-SKCM cohort was downloaded from the UCSC Xena platform. The proportion of CD8+ T cells was calculated by the CIBERSORT algorithm (32). Spearman’s rank correlation coefficient and regression line were calculated and visualized by ggscatter function in ggpubr R package.

### Differential Expression and Mutation Burden Analysis of the TCGA-SKCM Cohort

We calculated the TRS scores for all samples, and then divided them into two groups based on the median score. We used t-test to identify differentially expressed genes between the two groups. Genes with log2 fold change > 1.5 and FDR < 0.01 were considered as significantly differentially expressed. The functional enrichment analysis of differential genes was performed using Metascape (33). The somatic mutation profiles of the TCGA-SKCM cohort in the form of mutation annotation format (MAF) were obtained from the UCSC Xena platform (34), and analyzed by the maftools R package (35), including calculation of mutation burden, identification of frequently mutated genes and differentially mutated genes. The intratumor heterogeneity, Wound Healing, Homologous Recombination Defects, and Th17 Cells score of patients were downloaded from previous study (36).

### Assessing Relationship Between the TRS and Clinical Outcome of Melanoma Patients

We collected five cohorts of melanoma patients to assess the association of TRS with overall survival, including 2 cohorts from the GEO database under accessions GSE22153 (37) and GSE65904 (38), one cohort (Allen2015) kindly provided by the corresponding author (27), one cohort from the ENA database under accession ERP105482 (28), and the melanoma cohort from TCGA which was retrieved from the UCSC Xena platform (34). The median level of the GSVA scores of TRS in each cohort was used as the cutoff to stratify patients into two groups. Kaplan-Meier curves were used to visualize survival differences between the two groups, and log rank test was utilized to assess the significance.

### Refinement of the TRS Signature

In order to test whether all genes in the TRS were necessary to predict prognosis of melanoma patients, we performed stepwise Akaike’s Information Criterion (AIC) estimation to refine the TRS using the TCGA-SKCM cohort. In brief, we first calculated the original AIC for the univariate Cox regression model constructed based on the GSVA scores for the whole TRS. We then tried to remove each gene and re-calculated the AIC based on the GSVA scores of the remaining genes, and we finally discarded the gene resulting in the lowest AIC which was lower than the original AIC. In the next steps, we iteratively discarded one gene until the AIC didn’t decrease compared to the previous step.

### Comparison of Prognosis Prediction Performance With Published Prognostic Signatures In Melanoma

To assess the performance of the TRS, we compared it with the infiltration levels of CD8+ T cells and 8 published prognostic signatures of melanoma (39–46). The infiltration levels of CD8+ T cells in melanoma patients were calculated with the CIBERSORT algorithm (32). The risk scores of the 8 signatures were calculated as the summation of product of the coefficient, which were collected from the corresponding manuscripts, and the expression level of each gene in the signatures. Higher risk scores represented higher risk of poor survival probability. To keep consistency of the scores in prognosis prediction, we calculated the negative value of TRS scores and CD8+ T cell infiltration levels as the corresponding risk scores. The median level of each signature in each cohort was chosen as the cutoff to stratify patients into two groups. We compared the performance of these signatures in terms of hazard ratio, area under time-dependent ROC curves (AUC), concordance index (C-index) and restricted mean survival time (RMST) ratio. For each cohort, only signatures, among which all genes were detected, were considered.

### Statistical Analysis

Kaplan-Meier curves and forest plot were visualized using the survminer (47) package. Significance of survival differences between two groups of patients were determined by log rank tests. Time-dependent AUCs were calculated using the survivalROC (48) package. The hazard ratios and C-index were calculated with survival (49) package, and comparison of C-index was performed using compareC (50) package. The restricted mean survival time (RMST) ratio was estimated with survRM2 (51) package. The PLAGE, ssGSEA and zscore scoring algorithms were implemented in the GSVA package. All analyses were performed in R version 4.0.2.

## Results

### Tumor Infiltrating T Cells Exhibit Highly Heterogeneous Transcriptional States

**Figure 1:**
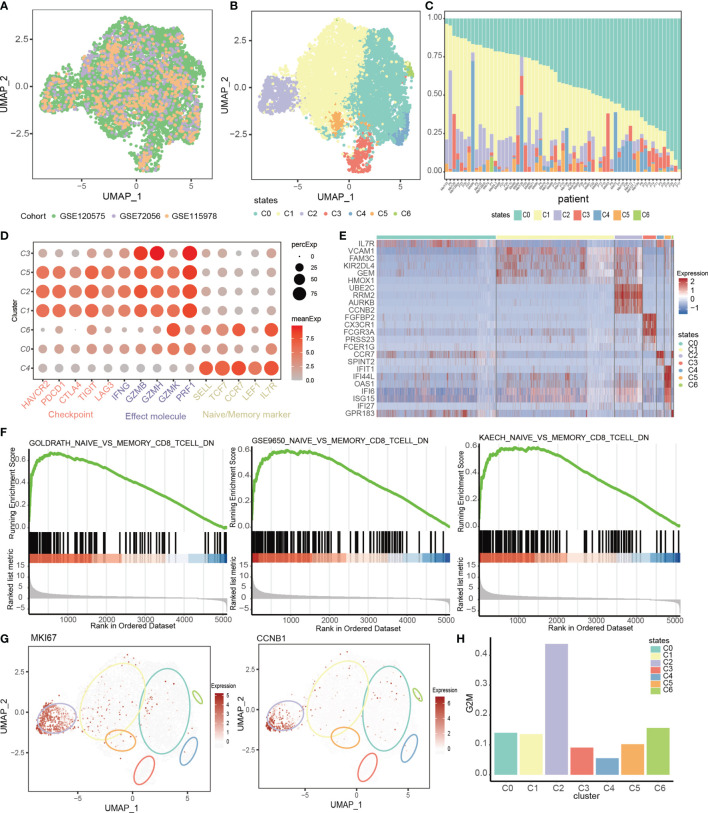
The T cell landscape of melanoma reveals transcriptionally heterogeneous cell states. **(A, B)** UMAP plots of CCA-integrated profiles of all CD8+ T cells used in this study colored by datasets **(A)** and by cell states **(B)**. **(C)** The composition of T cell states in each patient. **(D)** Dot plot showing distribution of checkpoints, effect molecules and naïve markers in each T cell state. Color denotes the average expression of each gene in each cluster. Circle size denotes the percentage of cells that expresses the gene within the indicated cluster. **(E)** Heatmap showing expression levels of differential genes in each cell state. **(F)** GSEA plot showing enrichment of the gene sets upregulated in memory CD8+ T cells in the cluster C0_memory T cells when compared to C4_naive T cells. **(G)** UMAP feature plot representation of cell cycle markers (MKI67, CCNB1) within individual T cell state. **(H)** The proportion of proliferative cells in each T cell state.

In order to explore the transcriptional heterogeneity of CD8+ T cells within tumor microenvironment, we downloaded three Smart-seq2 datasets [GSE120575 (16), GSE72056 (6) and GSE115978 (15)] of single-cell RNA sequencing (scRNA-seq) in melanoma from GEO database. A total of 8262 CD8+ T cells from 80 samples (see Methods, **Table S1**, and **Figures S1A–C**) were retained after initial quality control. Through canonical correlation analysis (CCA) (17), we integrated these three datasets with cohort information successfully corrected (**Figure 1A**). With graph-based clustering on the integrated expression profile, 7 cell clusters were identified (**Figure 1B**). Notably, we found that clusters C0 and C1 were the major population present in all patients (**Figure 1C**), suggesting the two clusters were shared among melanoma patients. Contrarily, other clusters had relatively small numbers of cells, and some were exclusively observed in a part of patients, which reflected the T cell heterogeneity among patients.

We characterized the identity of each cluster through differential gene identification of each cluster compared with all other T cells and by assessing the expression of well-known marker genes associated with T cell lineages and states (**Figures 1D, E**). We denoted the cluster C4 as the C4_naive state which specifically expressed naive marker genes such as *SELL*, *TCF7*, *CCR7* and *LEF1* (**Figure 1D**). The cluster C3 was characterized by *CX3CR1*, *PRF1*, *GZMH* and *GZMB*, which were related to the effector state of T cells, and by lack of co-inhibitory molecules (**Figure 1D**, **Figure S1D**), thus we denoted it as the C3_effect state. The cluster C6 was denoted as the C6_transition state as it moderately expressed naive markers and highly expressed *GZMK* (**Figure 1D**), which widely featured the intermediate state between naive and exhaustion T cell states (52). The cluster C0 was denoted as the C0_memory state due to the low levels of co-inhibitory and effector molecules and high level of *IL7R* (**Figure 1E**), which was associated with the memory state (53). Gene set enrichment analysis (GSEA) also revealed that the differentially expressed genes between cluster C0_memory and C4_naive were associated with CD8+ T cell memory signatures (**Figure 1F**). The remaining clusters were denoted as the C1_exhausted, C2_cellcycle and C5_interferon states because of the high levels of cytotoxic molecules and numerous inhibitory checkpoints, including *PDCD1*, *CTLA4* and *HAVCR2*, suggesting the exhausted states (54). In addition to the exhausted characteristics, the C2_cellcycle also had the highest levels of cell cycle markers and highest proportion of proliferative cells (**Figures 1G, H**, **Figure S1E**). And the cluster C5_interferon was characterized by the interferon signal and the signature genes of C5_interferon were involved in defense response to virus, response to interferon-gamma (**Figures S1F, G**).

### Exhausted CD8+ T Cells Exhibit Tumor Reactivity and Form Large TCR Clones

**Figure 2:**
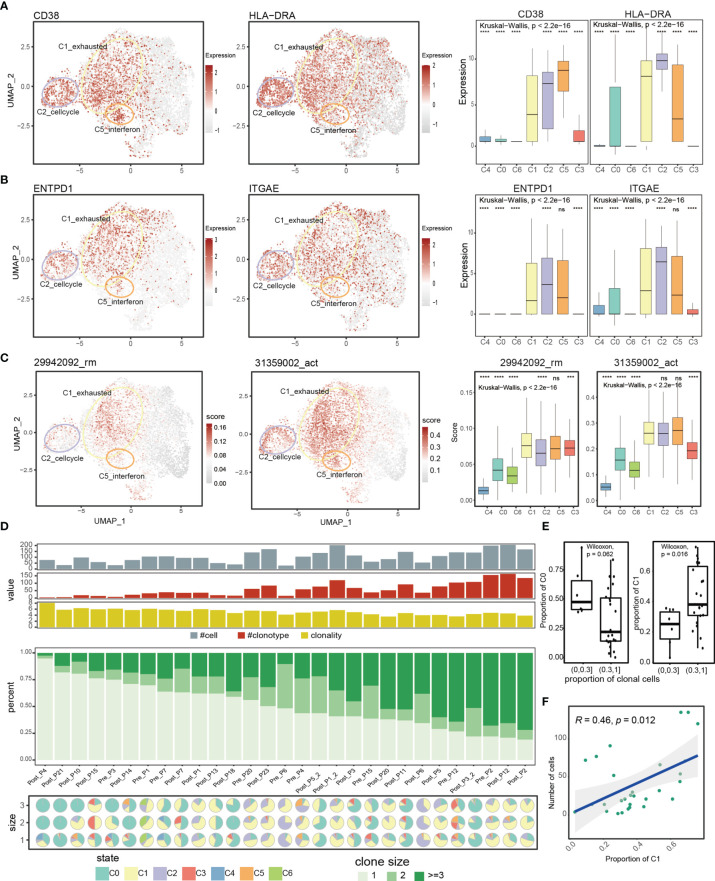
Comprehensive transcriptional analysis and T cell receptor analysis reveals enrichment of tumor-reactive CD8+ T cells in the C1_exhausted T cell state. **(A, B)** UMAP feature plot representation of T cell activation markers (CD38, HLA-DRA) and tumor reactive markers (ENTPD1, ITGAE) within individual T cell state. Boxplots showing the significance of the expression difference among T cell state. **(C)** UMAP feature plot representation of AUCell scores of T cell reside memory signatures and T cell activation signature within individual T cell state. Boxplots showing the significance of the expression difference among T cell state. **(D)** Clonal composition of T cells in each patient showing from top to bottom the number of T cells of which the TCR was retrieved, the number of clonotypes, the clonality score (defined by Shannon Entropy), the composition of clone size (size=1, size=2, and size>2), and the pie charts showing the cell states composition of each patient stratified by clone size. Patients are ordered by the proportions of size-one clones decreasingly. **(E)** The difference of cluster C0/C1 proportion between two sample groups, with high and low proportions of clonal cells. **(F)** Spearman correlation between the fraction of cells in C1_exhausted state and number of cells with TCRs across patients. ns denoted non-significant, *** denoted p < 0.001, and **** denoted p < 0.0001.

Recent studies have shown that tumor-reactive T cells exhibit exhausted phenotype (55–57). And the coupled expression of inhibitory molecules and effector molecules indicated that exhaustive T cells have not completely lost their effector functions. These motivated us to explore whether the exhausted clusters (C1_exhausted, C2_cellcycle and C5_IFNG) were enriched for tumor-reactive cells. We first assessed the expression of T cell activation markers *CD38* and *HLA-DRA* (58), and observed high expression of *CD38* and *HLA-DRA* in all of the exhausted clusters (**Figure 2A**), potentially reflecting recent antigen encounter. Furthermore, exhausted clusters specifically expressed tumor-reactive T cell markers (*ENTPD1* and *ITGAE*), while the effect cluster rarely expressed them (**Figure 2B**), indicating that cells belonging to the effect cluster were potentially bystander cells. In addition, we curated two tissue resident memory signatures (29942092_rm, 28930685_rm) and a T cell activation signature (31359002_act). The 29942092_rm is comprised of the differentially expressed genes of a tissue-resident memory T (TRM) cell cluster in breast cancer (8), the 28930685_rm is a core transcriptional and phenotypic signature which defines human tissue-resident memory for both CD4+ and CD8+ T cells (59), and the 31359002_act is a T cell activation signature, which consisted of the top 50 genes correlated with IFNG (60). We observed that the exhausted clusters displayed elevated levels of T cell activation signatures and tumor reside memory signatures (**Figure 2C**, **Figure S2A**), which were frequently observed in tumor-reactive T cells (61). These results indicated that cells in the exhausted clusters (C1_exhausted, C2_cellcycle and C5_IFNG) could potentially be tumor-reactive T cells.

T cell clonality has long been used as a marker of tumor reactivity (62). Previous studies have proven that the majority of TCR clones with high clonal expansion have been shown to be associated with tumor reactivity in melanomas (11, 63). In order to understand the T cell clonality across different clusters, we obtained the TCR sequences and explored the degrees of clonal expansion. We obtained TCR alpha and beta chains of 3078 T cells from Moshe et al. (16), with 1381 cells harboring unique TCRs, 500 cells harboring repeated TCRs, and 1197 cells with clonally expand TCRs (**Figure S2B**), with clonal size ranging from 3 to 59 (**Figure S2C**). Notably, different states exhibited different degrees of clonal expansion, with C0_memory state and C1_exhausted state showing relatively more clonal TCRs (**Figure S2D**). TCR clonotype composition were highly variable across patients, and different patients had different degrees of clonal expansion (**Figure 2D** middle panel). Patients with more clonal TCRs had higher proportion of cells in the C1_exhausted state, indicating the clonal expansion of exhausted state (**Figure 2D** down panel, **Figure 2E**). For instance, C1_exhauted T cells account for 74%, 69% and 82% of clonal TCR in patient Pre_P2, Post_P12, and Post_P2. On the contrary, memory state (C0) was dominant in patients with more unique TCRs (**Figure 2D** down panel, **Figure 2E**). Furthermore, cell proportion of the cluster C1 positively correlated with the number of T cells which the TCR was retrieved (**Figure 2F**), and correlated with the number of clonotypes (**Figure S2E**). In addition, we found samples with higher cluster C1 proportion have lower TCR diversity (**Figure S2E**), which was consistent with the previous study (64). These results highlighted that the C1_exhausted state was strongly enriched for larger clones and contributed to the TCR clonal expansion of patients. Collectively, given the high levels of T cell cytotoxicity, activation markers and signatures, and the greatest clonal expansion of C1_exhausted, we believed that the C1_exhausted state could reflect tumor reactivity.

### Identifying and Validating a Tumor-Reactive T Cell Signature

**Figure 3:**
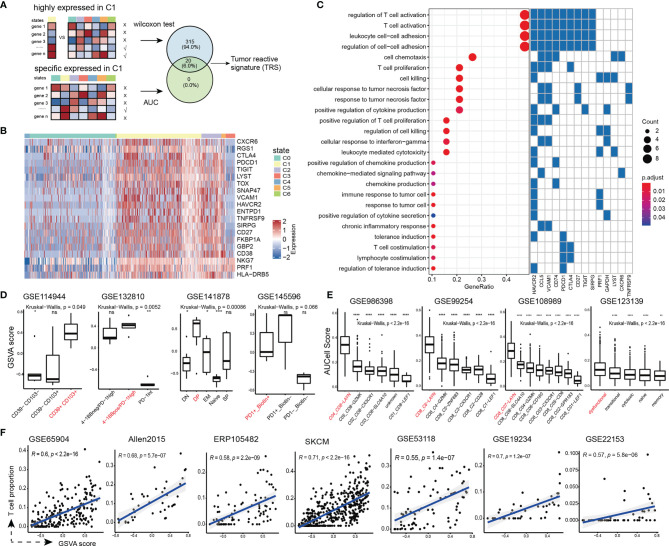
Development and validation of the TRS signature. **(A)** The framework to screen the TRS. **(B)** Heatmap showing the expression of the TRS in single cells. **(C)** (Left panel) Enriched GO terms of TRS. (Right panel) Genes involved in each GO term **(D)** Distribution of the TRS scores of tumor-reactive T cell group (colored by red) and other T cell groups. **(E)** Distribution of the TRS scores of T cell states in additional scRNA-seq datasets of liver cancer (GSE986398), non-small cell lung cancer (GSE99254), colorectal cancer (GSE108989) and melanoma (GSE123139). The exhausted T cell state is colored by red. Wilcoxon rank-sum tests were used to assess the significance of pairwise comparisons, and Kruskal-Wallis tests were used for overall comparisons. ns denoted non-significant, * denoted p < 0.05, ** denoted p <0.01, *** denoted p < 0.001, and **** denoted p < 0.0001. **(F)** Spearman correlation between the TRS scores and the proportions of T cell infiltration in bulk melanoma samples obtained from GSE65904, Allen2015, ERP105482, SKCM, GSE53118, GSE19234, and GSE22153.

To assess T cell reactive status of tumor samples, we attempted to develop a gene expression signature to specifically indicate tumor reactivity of T cells. Based on the integrated expression profile of all T cells, we applied Wilcoxon rank-sum test to identify the significantly highly expressed genes in the C1_exhausted state, and further employed the area under the ROC curve (AUC) to extract the specific genes which could efficiently distinguish the C1_exhausted state from the others, resulting in 20 genes (**Figure 3A**). These genes were defined as the tumor reactive signature (TRS), including co-inhibitory receptors (CTLA4, PDCD1, TIGIT and HAVCR2), reactive markers (CD38 and ENTPD1), effector molecules (NKG7 and PRF1), tumor necrosis factor TNFRSF9 and critical exhaustion-related regulator TOX (**Figure 3B**). The genes in TRS are widely involved in T cell activation, cell killing, response to tumor cell, chemokine production, cytokine secretion, and chronic inflammatory response (**Figure 3C**).

We performed multiple assessment to validate the performance and robustness of the TRS. First, we collected four independent gene expression datasets which contained samples enriched for tumor-reactive CD8+ T cells. We calculated the tumor reactivity score for each sample based on this 20-gene signature using the GSVA algorithm (21). Notably, almost in all cases, tumor-reactive group showed the highest tumor reactivity scores (**Figure 3D**), even calculating scores with different algorithms (**Figure S3A**). Second, we downloaded four additional scRNA-seq datasets, including hepatocellular carcinoma, non-small cell lung cancer, colorectal cancer and melanoma. We observed significantly higher tumor reactivity scores in exhausted states than the other T cell states (**Figure 3E** and **Figure S3B**), suggesting that the tumor-reactive signature was robust and widely present in a variety of cancer types. Third, we obtained seven bulk expression datasets of melanoma and estimated their infiltration levels of CD8+ T cells using the CIBERSORT algorithm (32). And we found that the TRS scores were highly correlated with proportions of CD8+ T cell infiltration (**Figure 3F**). These results demonstrated the robustness of the TRS to define tumor-reactive status in bulk tumor samples.

### Patients With High TRS Score Have Strong Immune Activity and High Mutation Burden

**Figure 4:**
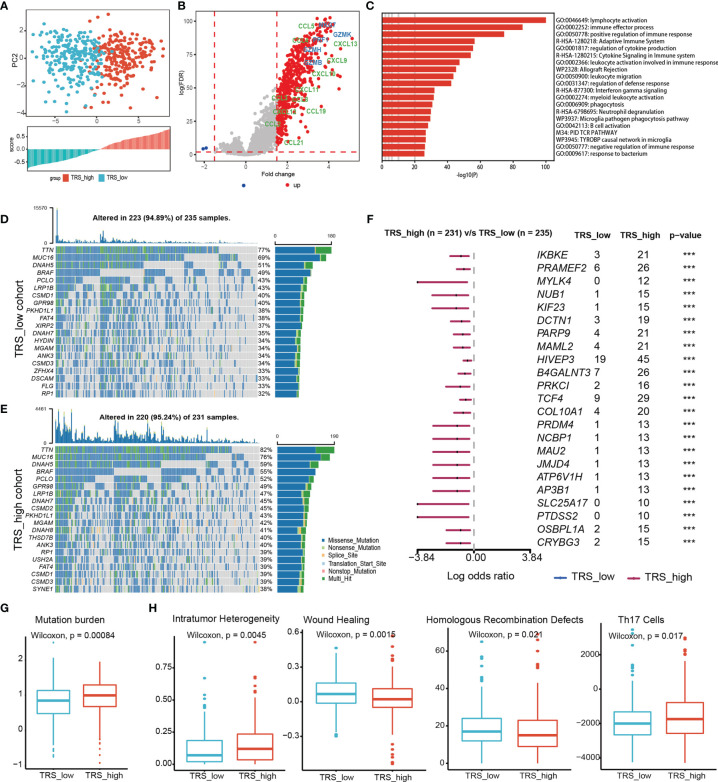
Comprehensive characterization of enriched pathways and genomic aberrations related to tumor reactivity. **(A)** (Up panel) Principle component analysis with the expression levels of TRS in SKCM. Barplot showing the TRS scores of SKCM patients. **(B)** Volcano plot showing differentially expressed genes between the TRS_high and TRS_low groups in TCGA cohort. Effect molecules were colored by blue, and chemokine were colored by green. **(C)** Barplots showing functional gene sets enriched in the significantly upregulated genes. **(D, E)** Top 20 most frequently mutated genes were illustrated in the TRS_low **(D)** and TRS_high **(E)** group. **(F)** Significantly differentially mutated genes between the two groups were displayed. Genes with p-value < 0.001 were considered as significant. **(G)** Boxplots showing differences of log transformed tumor mutation burden (TMB) between the TRS_high and TRS_low groups. **(H)** Boxplots showing differences of intratumor heterogeneity, Wound Healing, Homologous Recombination Defects, and Th17 Cells score between the TRS_high and TRS_low groups. *** denoted p < 0.001.

To characterize potential molecular mechanisms associated with tumor reactivity, we stratified melanoma patients from the TCGA-SKCM cohort into two groups according to the median GSVA scores of the TRS (**Figure 4A**). We first identified significantly differentially expressed genes between the two groups using t-test with log2 fold change > 1.5 and FDR < 0.01. Strikingly, differentially expressed genes were mostly up-regulated in the TRS-high group compared to the TRS-low group, including chemokines and cytotoxic-related genes (**Figure 4B**), indicating that patients in the TRS-high group were immune activated. We then performed functional enrichment analysis on the up-regulated genes through Metascape (33), and we did observe significant enrichment of pathways related to immune activation, such as lymphocyte activation, cytokine signaling in immune system, and inflammatory response (**Figure 4C**).

In order to identify mutations that primed T cell response to generate protective endogenous immunity against tumor, we comprehensively analyzed the mutation frequency of genes in patients with different TRS scores. Top 20 most frequently mutated genes in each group were displayed in **Figures 4D, E**. Although there were some overlap of top mutated genes between the two groups, we identified more frequently mutated genes in the TRS-high (**Figure 4F**), including melanosome-related gene AP3B1, apoptosis-related gene PRKC1, all of which showed the higher mutation frequencies in the group with higher TRS scores (**Figure 4F**). Moreover, there were higher tumor mutation burden in the TRS-high group (**Figure 4G**). In addition, we found that TRS_high group exhibited higher scores of intratumor heterogeneity and Th17 cell, and lower scores of wounding healing and homologous recombination defects (**Figure 4H**) (36).

### TRS Contributes to Longer Overall Survival Times in Melanoma Patients

**Figure 5:**
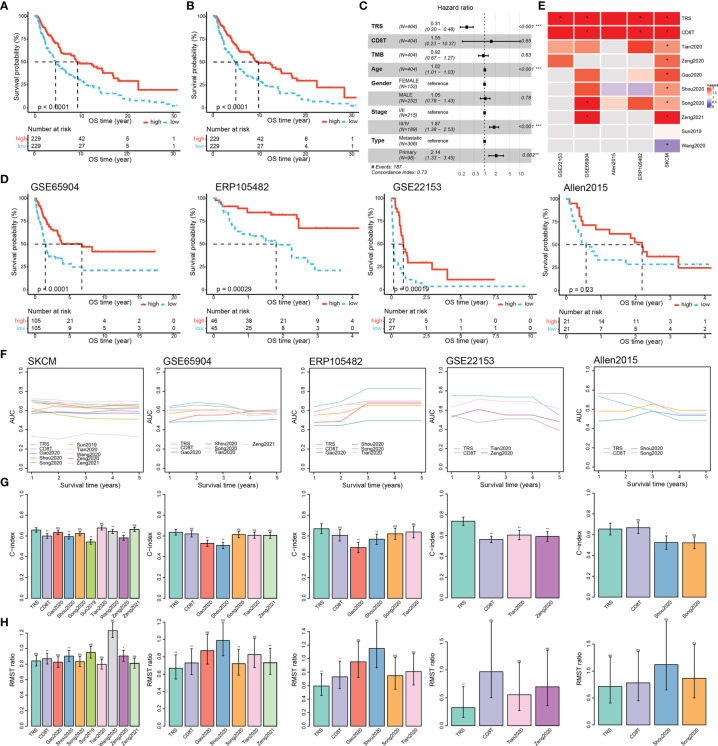
Prognostic assessment of the TRS in melanoma. **(A)** Kaplan-Meier survival curves showing significant differences of survival probabilities for patients stratification based on the median TRS score in the TCGA-SKCM cohort. **(B)** Kaplan-Meier survival curve analysis for the refined TRS. **(C)** Forest plot showing independent prognostic value of the TRS score adjusting for infiltration levels of CD8+ T cells and clinical characteristics in the TCGA-SKCM cohort. **(D)** Kaplan-Meier survival curves showing significant association of the TRS with overall survival in melanoma patients obtained from GSE65904, ERP105482, GSE22153 and Allen2015. **(E-H)** Comparison of prognostic performance of the TRS with CD8+ T cell infiltration and 8 published prognostic-related signatures in melanoma in terms of significance of patient stratification **(E)**, time-dependent AUC **(F)**, C-index **(G)** and restricted mean survival time (RMST) ratio between high-risk and low-risk groups **(H)**. In order to keep consistency of the scores in prognosis prediction, we calculated the negative value of TRS scores and CD8+ T cell infiltration levels as the corresponding risk scores. Colors in **(E)** denoted hazard ratios of the signatures in univariate Cox proportional hazard regression analysis, and * indicated significant stratification of melanoma patients in terms of survival probabilities based on the corresponding signatures. Comparisons of C-index between the TRS and the other signatures were performed using the compareC package **(G)**. ns denoted non-significant, * denoted p <0.05, ** denoted p <0.01, *** denotes p < 0.001.

We next attempted to explore the relationship between the TRS and the clinical outcomes of melanoma patients. We observed significant differences of survival probabilities between patient groups stratified based on the TRS scores using the TCGA-SKCM cohort (**Figure 5A**, log rank test, p-value < 0.0001). In order to examine robustness of the TRS, we performed stepwise AIC estimation (see Methods) and refined a 6-gene signature including *CTLA4*, *CXCR6*, *LYST*, *CD38*, *GBP2* and *HLA-DRB5*, which was enough for prognosis prediction (**Figure 5B**, log rank test, p-value < 0.0001). We also performed different scoring algorithms (including mean, PLAGE, ssGSEA and zscore) on the refined TRS to examine their impact on prognosis prediction. We demonstrated that the refined TRS could significantly stratify SKCM patients independent of the scoring algorithms (**Figures S4A–D**). Moreover, the refined TRS remained as an independent prognostic factor adjusting for CD8+ T cell infiltration levels, TMB and clinical characteristics, including age, gender, AJCC stage and metastatic status (**Figure 5C**). These indicated that prognostic value of the refined TRS was beyond the impact of CD8+ T cell infiltration or TMB despite their positive correlation. Furthermore, we verified the prognostic association of the refined TRS in another 4 cohorts of melanoma patients (**Figure 5D**, log rank tests, p-values <0.0001, = 0.00029, = 0.00019 and =0.23 for GSE65904, ERP105482, GSE22153 and Allen2015, respectively). Although it was not significant enough for Allen2015 due to the limit sample size, we observed obvious distinction of survival probabilities between two groups stratified by the refined TRS.

To further estimate the performance of the refined TRS on prognosis prediction, we collected 8 published gene signatures (39–46) and compared their performance in the five cohorts. To keep consistency of the scores in prognosis prediction, we calculated the negative value of TRS scores and CD8+ T cell infiltration levels as the corresponding risk scores. For the 8 published signatures, we calculated their risk scores as summation of the product of coefficient and expression level of each gene, which was exactly as described in the corresponding study. Notably, TRS was the only one which showed consistent risk trend and significant stratification of melanoma patients in terms of overall survival (**Figure 5E**). In addition, TRS was among the top-performance signatures in all cohorts in terms of time-dependent AUCs, C-index and RMST ratio (**Figures 5F–H**). We also calculated GSVA scores for the 8 published signatures, which was the same scoring algorithm for the TRS. The results still displayed higher and consistent performance of the refined TRS than the other signatures (**Figures S4E–H**). These results suggested that the TRS was widely applicable and achieved consistently high performance in multiple cohorts in different platforms.

### Tumor Reactive Signature Predicts Immunotherapy Response

**Figure 6:**
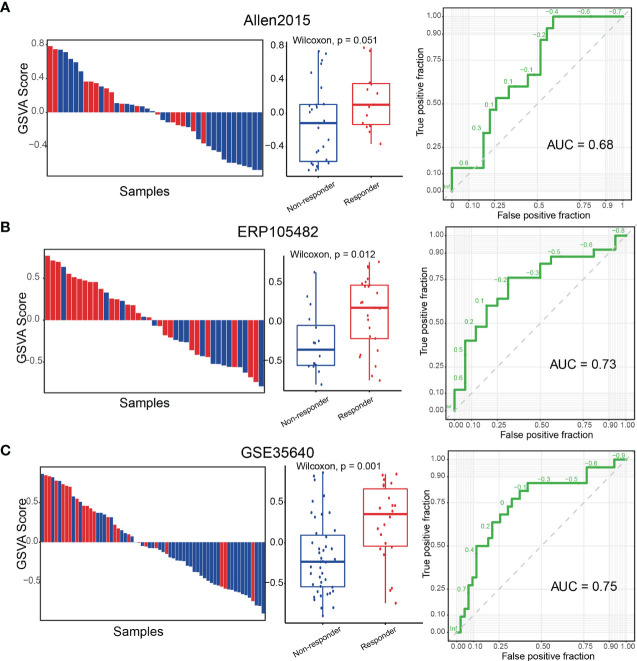
The TRS score predicts response to immunotherapy in melanoma. **(A)** (Left panel) Waterfall plot of the TRS scores depicting immunotherapy response of melanoma patients from the Allen2015 cohort. (Middle panel) Boxplots showing differences of the TRS scores between responders and non-responders. (Right panel) ROC curves for the performance of TRS in predicting response to immunotherapy. **(B, C)** Same as **(A)** for the ERP105482 cohort **(B)** and the GSE35640 cohort **(C)**.

ICB therapies were designed to reinvigorate efficacious anti-tumor immune responses by targeting inhibitory receptors on T cells. We noted that a canonical immune checkpoint molecules CTLA4 (65) were included in the refined TRS. Therefore, we next examined whether the refined TRS could predict ICB clinical response utilizing two cohorts (Allen2015 and ERP105482) of melanoma patients treated with anti-PD1 or anti-CTLA4 ICB therapies. We first investigated the relationship between the refined TRS scores and the expression of ICB therapy targets (**Figure S5**), and observed significant correlation between them, indicating the potential of the TRS to predict immunotherapy response. In the two cohorts, all patients were classified as responders or non-responders according to the RECIST criteria. Our results showed that the responders had significantly higher TRS scores than the non-responders (**Figure 6**). Finally, receiver operating characteristic curve analysis of the TRS scores in predicting response to ICB therapies yielded high performance (AUC = 0.68 and 0.73 for Allen2015 and ERP105482, respectively). In addition, we also evaluated the predictive performance of the refined TRS in a cohort (GSE35640) of patients received the MAGE-A3 immunotherapy. Similarly, patients with higher TRS scores exhibited higher proportions of responders, and the AUC of predicting response to MAGE-A3 therapy reached 0.75 (**Figure 6**). In summary, these results suggested that the TRS scores could be used to predict response to immunotherapies of melanoma patients.

## Discussion

In this study, we integrated three scRNA-seq datasets of T cells in melanoma, and identified a subgroup of tumor-reactive T cells. A 22-gene signature (TRS) was developed and validated to evaluate the degree of T cell reactivity to tumor cells in melanoma patients. Applying TRS to the TCGA-SKCM cohorts, we characterized the pathways and mutations related to tumor reactivity. Next, through analyzing the TRS scores in multiple cohorts, we validated that the tumor-reactive signature could act as an independent prognostic factor for overall survival of melanoma patients and a predictor for the response to cancer immunotherapy.

Infiltration of T cells were conventionally thought to correlate with better survival of tumor patients. However, the association between tumor infiltrating T cells and tumor-reactive T cells were confounded by the bystander and non-tumor-reactive T cells. Therefore, it was important to efficiently identify and estimate infiltration levels of the tumor-reactive T cells in tumor patients. A previous study showed that the strength of exhausted signature of melanoma was positively correlated with the presence of tumor-reactive T cells, while the strength of cytotoxic signature was negatively correlated with it (19). Consistent with this study, our results demonstrated that the tumor-reactive T cells were enriched in the exhausted T cell compartment, which exhibited specific expression of tumor-reactive markers and TCR clonal expansion. While the cytotoxic effect T cells were considered as bystanders, which might be activated by viruses (66). In addition, T cell clonality and expression of CD39 and CD103 were used as markers of tumor reactivity (55). Interestingly, we did observe TCR clonal expansion of T cells in the C1_exhausted state, which we believed to be the tumor-reactive T cell cluster.

Although T cell clonality and expression of CD39 and CD103 were thought to reflect tumor reactivity, using them alone was not robust to identify tumor-reactive T cells. For instance, TCR expanded T cells which did not reactive against cancer cells were also observed in the tumor microenvironment (11), and CD39- T cells also showed the ability to kill cancer cells (67). Therefore, it was important to develop a robust and efficient signature to identify tumor-reactive T cells, which was beneficial for patient stratification in clinical management such as prognosis and cancer immunotherapy. In our study, we developed a 22-gene signature which we called the TRS. In addition to tumor reactive markers, the TRS also consisted of multiple factors related to tumor reactivity, including co-inhibitory receptors, T cell activation markers and effect molecules. The combination of these genes enhanced the robustness of the TRS in evaluating the strength of tumor reactivity, which were verified in multiple cohorts (**Figures 3C–E**). In addition, we utilized alternative scoring methods to calculate the TRS scores both for single-cell data and bulk data. The results showed a high degree of consistency, further demonstrating the robustness of the signature.

Current knowledge of tumor-immune ecosystem has allowed a rational stratification of patients based on the Immunoscore, which is a robust, consensus, and standardized scoring system of lymphocyte populations (68). According to the Immunoscore, tumors can be classified into “hot” (highly infiltrated, T cell inflamed) or “cold” (non-infiltrated, non-T cell-inflamed). In our study, the TRS scores were highly correlated with the levels of T cell infiltration. Patients with high TRS scores corresponded to hot tumors (69), demonstrating strong immune activity such as T cell activation, inflammatory response and cytokine production, and exhibited better survival. However, the prognostic value of the TRS was not merely reflection of T cell infiltration, as we demonstrated higher performance of the TRS than infiltration levels of CD8+ T cells, in multiple melanoma cohorts. Furthermore, we demonstrated better performance and robustness of the TRS in prognosis prediction than previously published prognostic signatures in melanoma. Moreover, we also demonstrated significant association of the TRS scores with response to immunotherapy of melanoma patients.

In summary, we identified a subset of exhausted T cells enriched for tumor-reactive T cells, and developed and validated a tumor-reactive signature to evaluate the tumor reactivity. Through comprehensive analyses of multiple independent cohorts, we proved that the TRS scores could be used to predict prognosis and immunotherapy response. In order to apply the TRS to predict prognosis or response to immunotherapy, we recommend to use the TPM-normalized log-transformed expression profile for bulk RNA-seq data, and RMA-normalized log-transformed profile for microarray data. Then, GSVA could be implemented to evaluate the enrichment scores of the refined TRS with default parameters. Alternatively, other algorithms such as PLAGE, ssGSEA, zscore and even simply mean expression could also be employed to calculate the TRS scores. Patients with higher TRS scores could have better survival and better response rate to immunotherapy. While for the PLAGE scores, higher scores may not indicate higher activities as PLAGE calculates the first principal component as the gene-set score. We could also apply the TRS to indicate potential tumor-reactive T cells. In this case with single-cell data, we recommend to use the Seurat R package to filter noise and low-quality cells, and then use AUCell or VISION tools to calculate TRS scores for CD8+ T cells. Cells with higher scores could potentially be tumor-reactive.

## Data Availability Statement

Publicly available datasets were analyzed in this study. The names of the repositories and accession numbers can be found in the **Supplementary Material**. The three single cell RNA-seq datasets used for cell states analysis were all downloaded from GEO database (https://www.ncbi.nlm.nih.gov/geo/) under accession numbers GSE120575, GSE115978 and GSE72056. The 12 datasets used for validation of TRS signature were downloaded from GEO database under accession numbers GSE98638, GSE99254, GSE108989, GSE123139, GSE114944, GSE132810, GSE141878, GSE145596, GSE19234, GSE53118, GSE22153 and GSE65904, and from the UCSC Xena platform (https://xenabrowser.net/datapages/) under accession TCGA Melanoma (SKCM). Three datasets were used for predicting the response to immunotherapy, including ERP105482 from ENA (https://www.ebi.ac.uk/ena/browser/home), GSE35640 from GEO (https://www.ncbi.nlm.nih.gov/geo/), and the dataset Allen2015 which was kindly provided by the corresponding author (PMID: 26359337).

## Author Contributions

XL, YX and YPZ conceived and designed the study. MY, JH and YYP designed the framework. MY, JH, LWX and GML analyzed the data and implemented the methodology. ZDJ and BP revised the manuscript. MY and SQS acquired the data. JH and YYP organized figures. GML and LWX provided constructive discussions. MY and JH helped in interpreting the results. MY, JH and YYP drafted the manuscript. All authors read and approved the manuscript.

## Funding

This work was supported in part by the National Key R&D Program of China (2018YFC2000100), the National Natural Science Foundation of China (61873075, 32070673, 31871336 and 31900478), HMU Marshal Initiative Funding (HMUMIF-21008), the Heilongjiang Provincial Natural Science Foundation (YQ2019C012), the Heilongjiang Postdoctoral Foundation (LBH-Q18099) and the Program for Young Scholars with Creative Talents in Heilongjiang Province (UNPYSCT-2017059).

## Conflict of Interest

The authors declare that the research was conducted in the absence of any commercial or financial relationships that could be construed as a potential conflict of interest.

## Publisher’s Note

All claims expressed in this article are solely those of the authors and do not necessarily represent those of their affiliated organizations, or those of the publisher, the editors and the reviewers. Any product that may be evaluated in this article, or claim that may be made by its manufacturer, is not guaranteed or endorsed by the publisher.
