# Supplementary material for: Single-Cell Transcriptomic Analysis Reveals a Tumor-Reactive T Cell Signature Associated With Clinical Outcome and Immunotherapy Response In Melanoma
Source: Front Immunol. 2021 Nov 5;12:758288. doi: 10.3389/fimmu.2021.758288 (PMC8602834; doi:10.3389/fimmu.2021.758288)
Supplement: Supplementary file 1 [file DataSheet_1.docx]

**
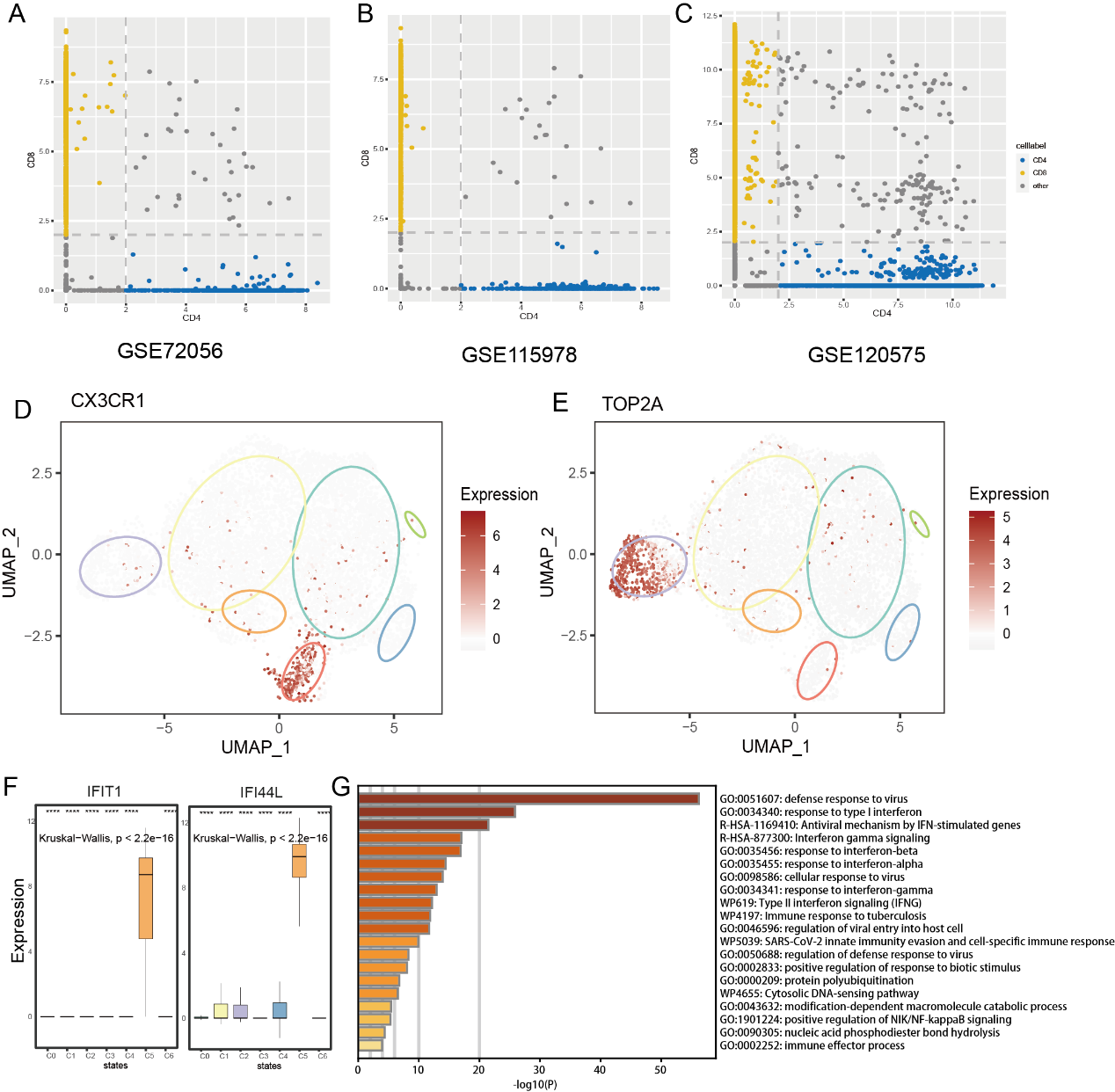
**

**Figure S1. Characterization of cell states of CD8+ T cells.** (A-C) Expression distribution of CD4 and CD8 in cells derived from GSE72056 (A), GSE115978 (B) and GSE120575 (C). (D-E) UMAP plot representation of CD8+ T cell states with color coded by expression levels of CX3CR1 and TOP2A. (F) The differential expression of interferon gene IFIT1 and IFI44L among the seven clusters. (G) Functional enrichment analysis of differentially expressed genes in the C5_IFNG state.


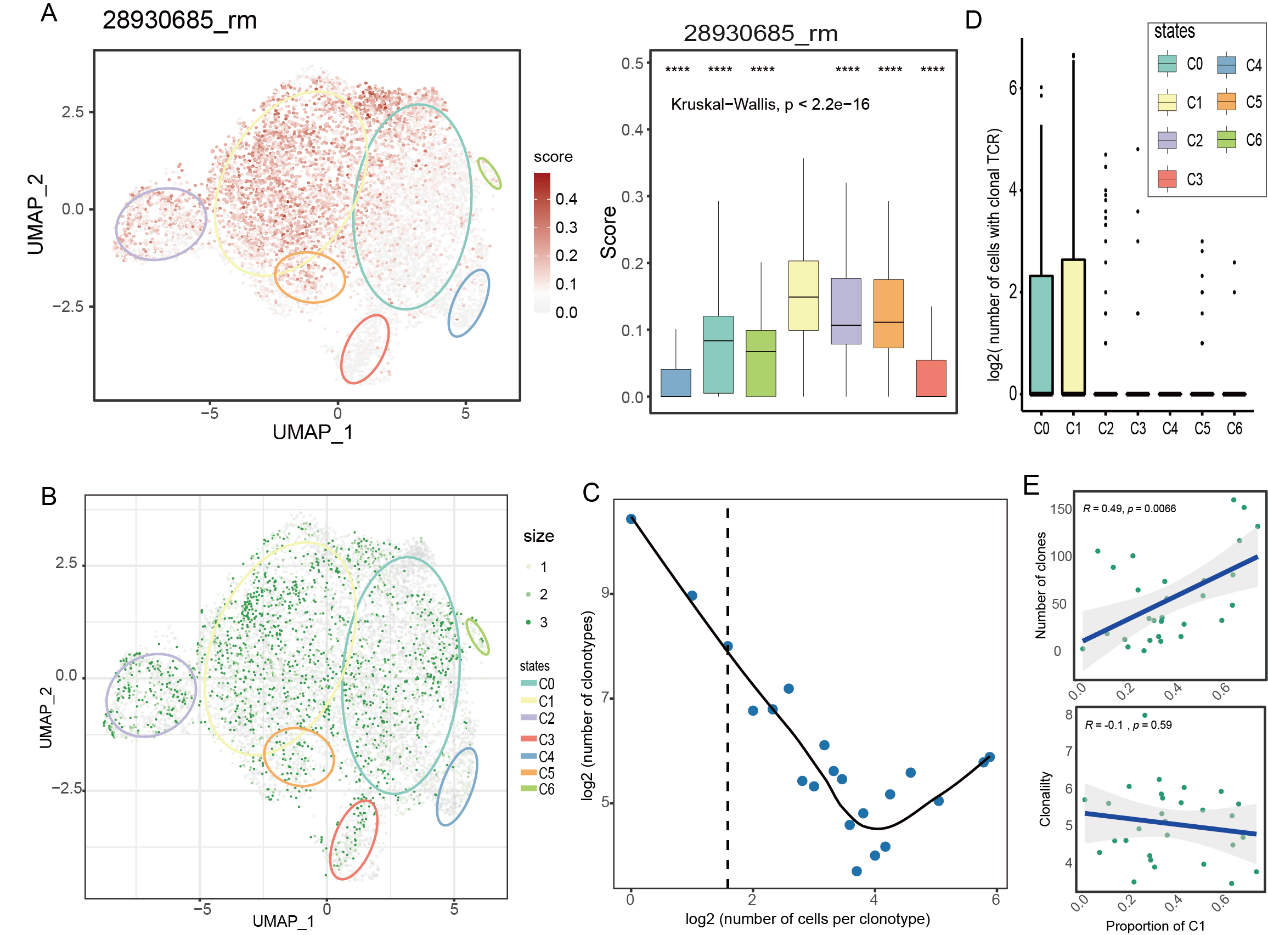


**Figure S2. TCR clone size distribution of CD8+ T cells.** (A) UMAP feature plot representation of AUCell scores of T cell reside memory signatures within individual T cell state. Boxplots showing the significance of the expression difference among T cell state. (B) UMAP plot representation of TCR clone size. (C) The association between the number of TCR clonotypes and the number of cells per clonotype. (D) The number of cells with clonal TCR in each cluster. (E) Spearman correlation between the fraction of cells in C1_exhausted state and the number TCR clones, clonality score.


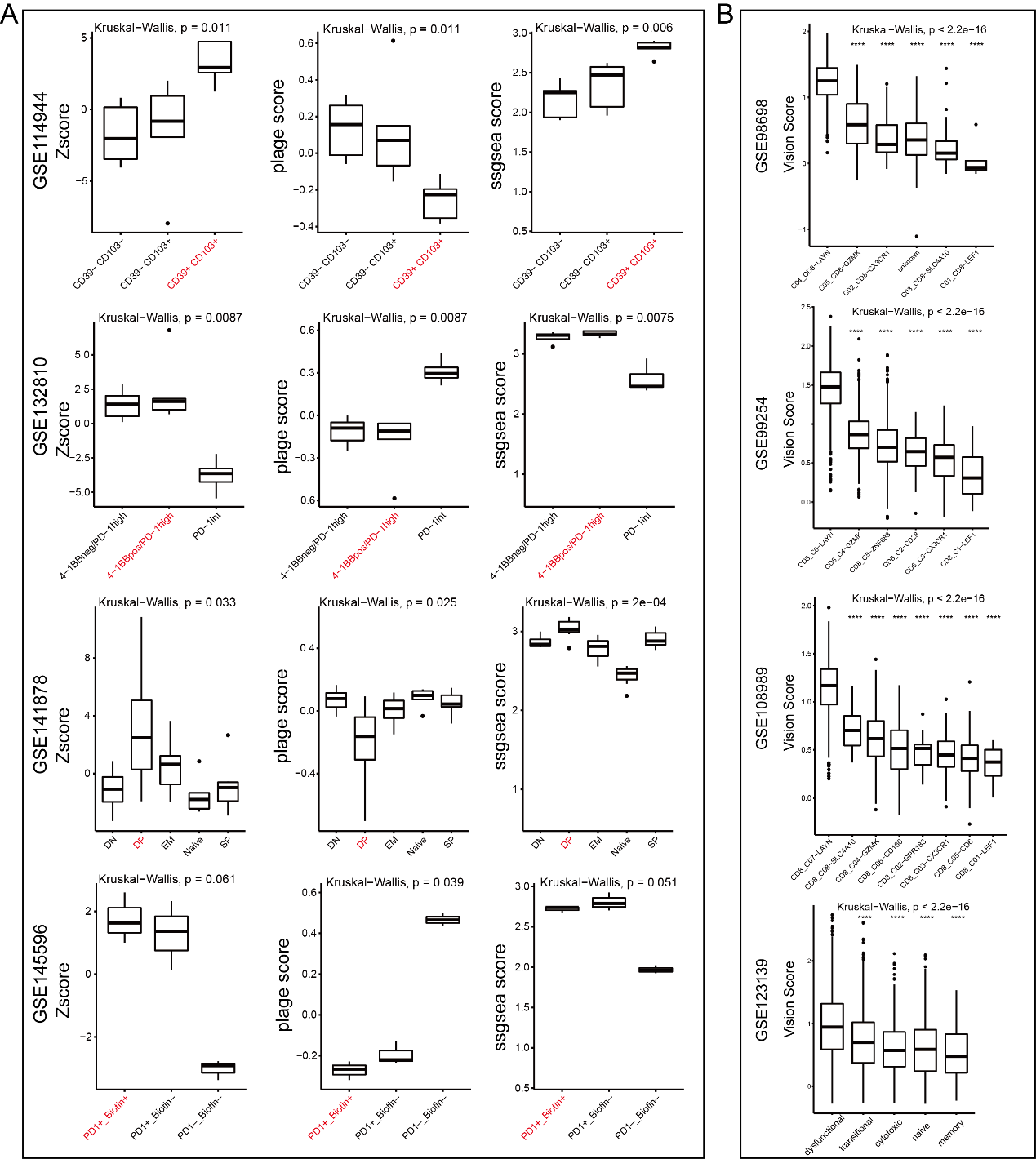


**Figure S3. TRS scores based on alternative scoring algorithms for validation datasets.** (A) Zscore, plage, and ssgsea scores for tumor reactive datasets. For the PLAGE scores, we observed opposite directions compared to the other methods as PLAGE calculated the first principal component as the gene-set score where higher scores may also indicate lower activities. (B) Vision scores for four single cell datasets.


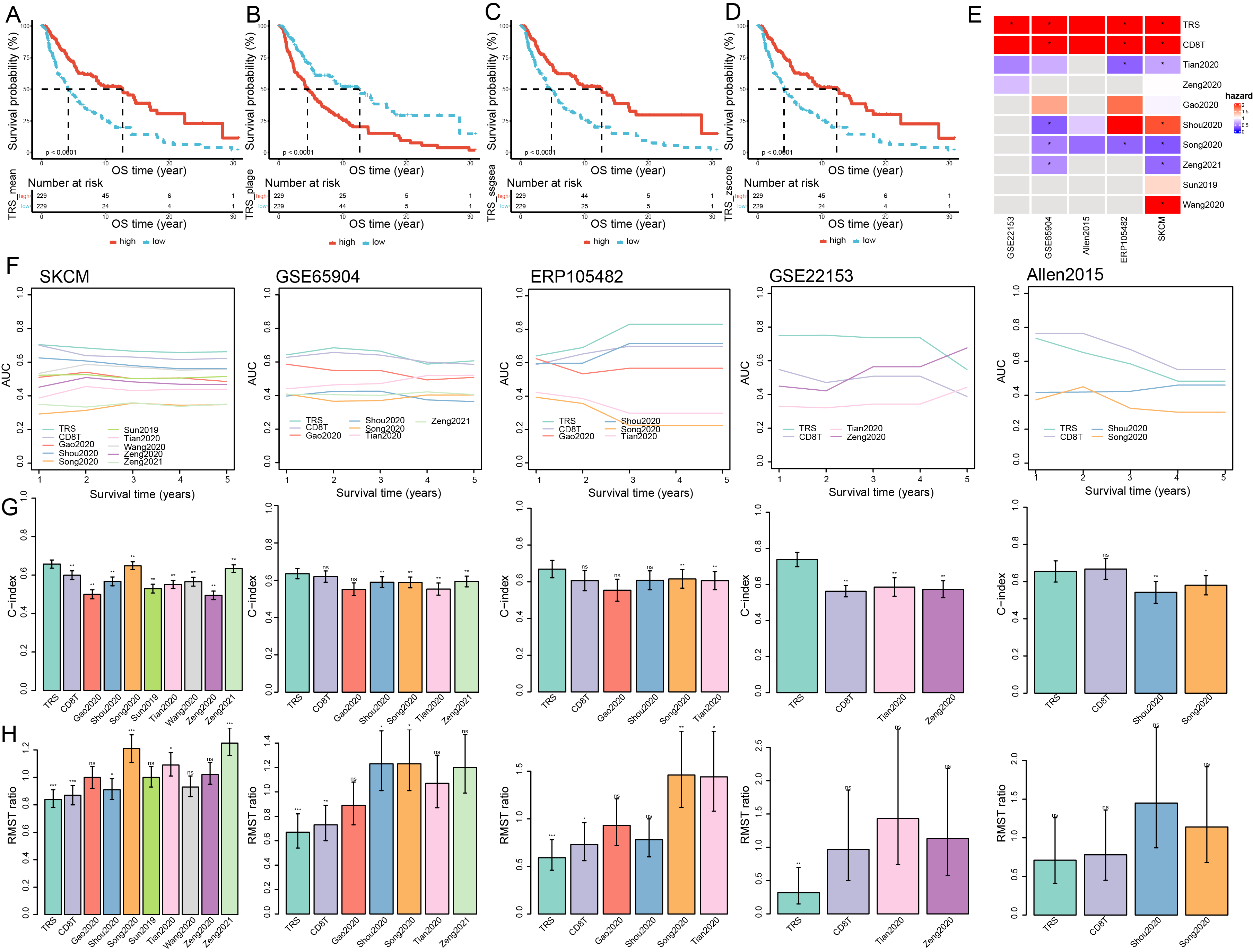


**Figure S4. Comparison of prognostic performance with other signatures with the GSVA scoring algorithm.** (A-D) Kaplan-Meier survival curves for patient stratification based on the refined TRS scores calculated by mean (A), PLAGE (B), ssGSEA (C) and zscore (D). For the PLAGE scores, we observed opposite directions compared to the other methods as PLAGE calculated the first principal component as the gene-set score where higher scores may also indicate lower activities. (E-H) Comparison of prognostic performance of the TRS with CD8+ T cell infiltration and 8 published prognostic-related signatures in melanoma based on the GSVA scores in terms of significance of patient stratification (E), time-dependent AUC (F), C-index (G) and restricted mean survival time (RMST) ratio between high-risk and low-risk groups (H). In order to keep consistency of the scores in prognosis prediction, we calculated the negative value of TRS scores and CD8+ T cell infiltration levels as the corresponding risk scores. Colors in (E) denoted hazard ratios of the signatures in univariate Cox proportional hazard regression analysis, and * indicated significant stratification of melanoma patients in terms of survival probabilities based on the corresponding signatures. Comparisons of C-index between the TRS and the other signatures were performed using the compareC package (G)


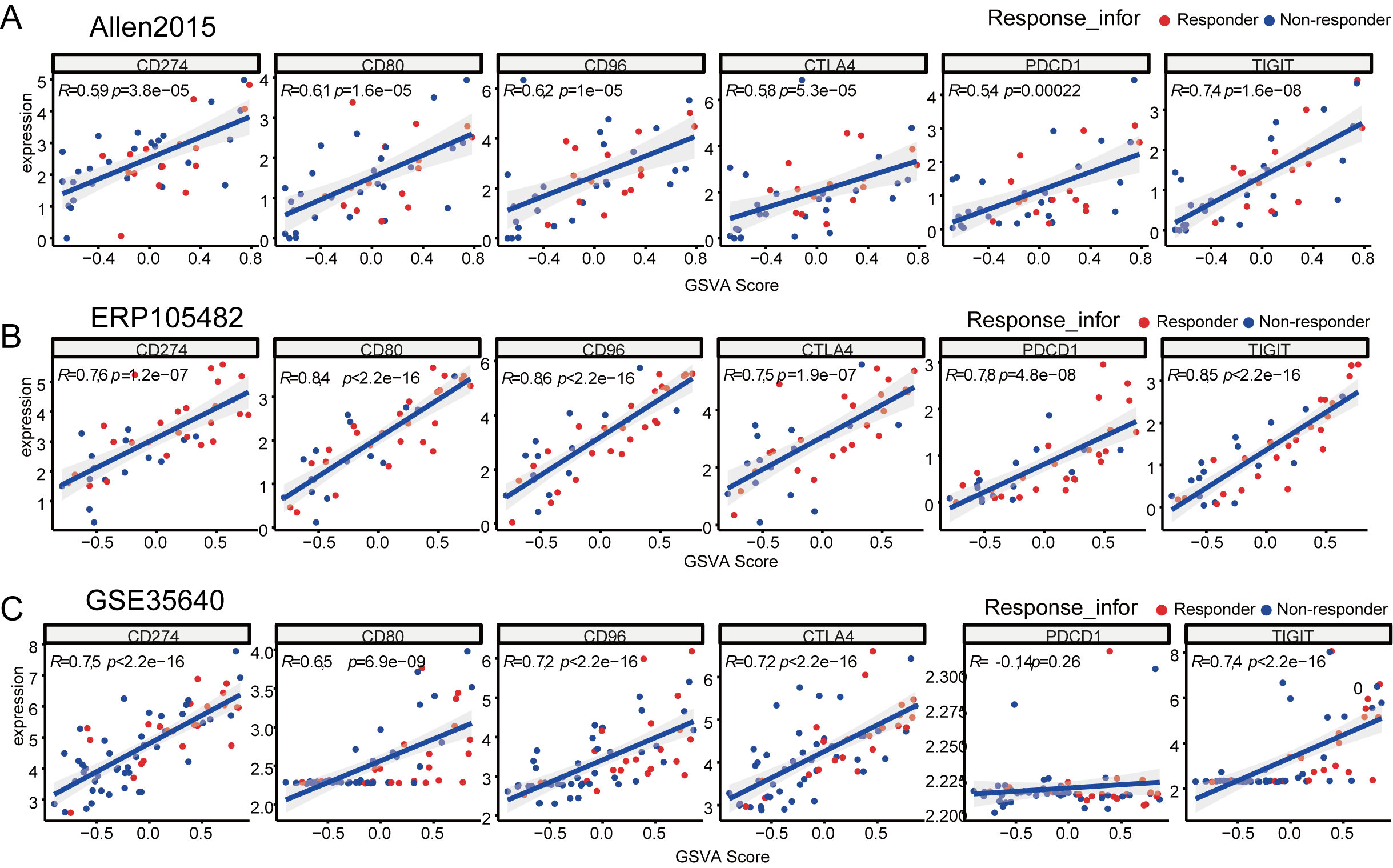


**Figure S5. Correlation between the TRS scores and the expression of immunotherapy target genes.** (A-C) Spearman correlation between the TRS scores and the expression levels of CD274, CD80, CD96, CTLA4, PDCD1 and TIGIT in ERP105482 (A), Allen2015 (B) and GSE35640 (C).

**Table S1. Datasets used in this study.**

| **Dataset** | **usage** | | | | | | | | | | **Cancer type­** | **Protocol** | **PMID** | **repositories** |
| --- | --- | --- | --- | --- | --- | --- | --- | --- | --- | --- | --- | --- | --- | --- |
|  | **A** | **B** | **C** | **D** | **E** | **F** | **G** | **H** | **I** | **J** |  |  |  |  |
| GSE120575 | **√** | **√** | **√** |  |  |  |  |  |  |  | melanoma | Smart-seq2 | 30388456 | GEO |
| GSE115978 | **√** |  | **√** |  |  |  |  |  |  |  | melanoma | Smart-seq2 | 30388455 | GEO |
| GSE72056 | **√** |  | **√** |  |  |  |  |  |  |  | melanoma | Smart-seq2 | 27124452 | GEO |
| GSE98638 |  |  |  | **√** |  |  |  |  |  |  | HCC | Smart-seq2 | 28622514 | GEO |
| GSE99254 |  |  |  | **√** |  |  |  |  |  |  | NSCLC | Smart-seq2 | 29942094 | GEO |
| GSE108989 |  |  |  | **√** |  |  |  |  |  |  | CRC | Smart-seq2 | 30479382 | GEO |
| GSE123139 |  |  |  | **√** |  |  |  |  |  |  | melanoma | MARS-seq2.0 | 30595452 | GEO |
| GSE114944 |  |  |  |  | **√** |  |  |  |  |  | HNSCC | Microarray | 30006565 | GEO |
| GSE132810 |  |  |  |  | **√** |  |  |  |  |  | HCC | RNA-seq | 31353502 | GEO |
| GSE141878 |  |  |  |  | **√** |  |  |  |  |  | CRC | RNA-seq | 31892342 | GEO |
| GSE145596 |  |  |  |  | **√** |  |  |  |  |  | CRC (murine) | RNA-seq | 33096019 | GEO |
| GSE19234 |  |  |  |  |  | **√** |  |  |  |  | melanoma | Microarray | 19915147 | GEO |
| GSE53118 |  |  |  |  |  | **√** |  |  |  |  | melanoma | Microarray | 22931913 | GEO |
| TCGA-SKCM |  |  |  |  |  | **√** |  |  | **√** | **√** | melanoma | RNA-seq | TCGA | TCGA |
| GSE22153 |  |  |  |  |  | **√** | **√** |  |  |  | melanoma | Microarray | 20460471 | GEO |
| GSE65904 |  |  |  |  |  | **√** | **√** |  |  |  | melanoma | Microarray | 25909218 | GEO |
| Allen2015 |  |  |  |  |  | **√** | **√** | **√** |  |  | melanoma | RNA-seq | 26359337 | Provided by the corresponding author |
| ERP105482 |  |  |  |  |  | **√** | **√** | **√** |  |  | melanoma | RNA-seq | 30753825 | ENA |
| GSE35640 |  |  |  |  |  |  |  | **√** |  |  | melanoma | Microarray | 23715562 | GEO |

**Usage:**

A: Cell state analysis

B: TCR analysis

C: Development of TRS

D: Validation of TRS for higher levels in exhausted cell state

E: Validation of TRS for higher levels in tumor-reactive cell group

F: Validation of TRS for correlation with infiltration of CD8+ T cells

G: Assessment of prognostic value for TRS

H: Assessment of prediction of response to immunotherapy

I: Differential expression analysis;

J: Mutation analysis;

**Cancertype:**

HCC: hepatocellular carcinoma

NSCLC: non-small cell lung cancer

CRC: colorectal cancer

HNSCC: head and neck cancer
